# Profiling Ribonucleotide and Deoxyribonucleotide Pools Perturbed by Remdesivir in Human Bronchial Epithelial Cells

**DOI:** 10.3389/fphar.2021.647280

**Published:** 2021-05-04

**Authors:** Yan Li, Hui-Xia Zhang, Wen-Di Luo, Christopher Wai Kei Lam, Cai-Yun Wang, Li-Ping Bai, Vincent Kam Wai Wong, Wei Zhang, Zhi-Hong Jiang

**Affiliations:** ^1^ State Key Laboratory of Quality Research in Chinese Medicines, Macau Institute for Applied Research in Medicine and Health, Macau University of Science and Technology, Guangdong-Hong Kong-Macao Joint Laboratory of Respiratory Infectious Disease (Macau University of Science and Technology), Taipa, Macau, China; ^2^ Faculty of Medicine and State Key Laboratory of Quality Research in Chinese Medicines, Macau University of Science and Technology, Taipa, Macau, China

**Keywords:** remdesivir, perturbation of nucleotide pools, inhibition of RNA and DNA synthesis, inhibition of CTP synthase, cell cycle arrest, Covid-19 therapy

## Abstract

Remdesivir (RDV) has generated much anticipation for its moderate effect in treating severe acute respiratory syndrome coronavirus 2 (SARS-CoV-2) infection. However, the unsatisfactory survival rates of hospitalized patients limit its application to the treatment of coronavirus disease 2019 (COVID-19). Therefore, improvement of antiviral efficacy of RDV is urgently needed. As a typical nucleotide analog, the activation of RDV to bioactive triphosphate will affect the biosynthesis of endogenous ribonucleotides (RNs) and deoxyribonucleotides (dRNs), which are essential to RNA and DNA replication in host cells. The imbalance of RN pools will inhibit virus replication as well. In order to investigate the effects of RDV on cellular nucleotide pools and on RNA transcription and DNA replication, cellular RNs and dRNs concentrations were measured by the liquid chromatography-mass spectrometry method, and the synthesis of RNA and DNA was monitored using click chemistry. The results showed that the IC_50_ values for BEAS-2B cells at exposure durations of 48 and 72 h were 25.3 ± 2.6 and 9.6 ± 0.7 μM, respectively. Ten (10) μM RDV caused BEAS-2B arrest at S-phase and significant suppression of RNA and DNA synthesis after treatment for 24 h. In addition, a general increase in the abundance of nucleotides and an increase of specific nucleotides more than 2 folds were observed. However, the variation of pyrimidine ribonucleotides was relatively slight or even absent, resulting in an obvious imbalance between purine and pyrimidine ribonucleotides. Interestingly, the very marked disequilibrium between cytidine triphosphate (CTP) and cytidine monophosphate might result from the inhibition of CTP synthase. Due to nucleotides which are also precursors for the synthesis of viral nucleic acids, the perturbation of nucleotide pools would block viral RNA replication. Considering the metabolic vulnerability of endogenous nucleotides, exacerbating the imbalance of nucleotide pools imparts great promise to enhance the efficacy of RDV, which possibly has special implications for treatment of COVID-19.

## Introduction

Remdesivir (RDV), an adenine nucleotide analog when inserted into viral RNA chains results in their premature termination (Warren et al., 2016), has shown a broad spectrum of antiviral activity against severe acute respiratory syndrome coronavirus (SARS–CoV) (Sheahan et al., 2017), Nipah virus (Lo et al., 2019), Middle East respiratory syndrome coronavirus (MERS–CoV) (de Wit et al., 2020; Sheahan et al., 2020), Ebola virus (Jacobs et al., 2016; Dornemann et al., 2017; Mulangu et al., 2019), and SARS-CoV-2 (Beigel et al., 2020; Holshue et al., 2020; Wang et al., 2020a). Because of the advantage of RDV in shortening the time for recovery in adults infected with SARS-CoV-2, the US Food and Drug Administration issued an Emergency Use Authorization for the use of remdesivir for the treatment of hospitalized patients with coronavirus disease 2019 (COVID-19) (US Food and Drug Administration, 2020). Based on the previous studies, RDV exerts its antivirus activity by specifically inhibiting the activity of viral RNA-dependent RNA polymerases (RdRps), which are crucial to virus survival not only through replication but also as engines of genome variability and evolution, without interference with human RNA polymerase (Tchesnokov et al., 2019; Ju et al., 2020).

Successful applications of metabolic reprogramming to treat cancer (Luengo et al., 2017) and inflammation (Ip et al., 2017) have prompted us to explore the potentials of RDV treatment. Like other nucleotide analogues, RDV is subjected to phosphorylation to form bioactive triphosphate, which is substrate-competitive with ATP for incorporation by viral RdRp and inhibition of viral RNA synthesis (Ray et al., 2016; Lee et al., 2017). The Phosphoramidate (ProTide) approach is used to establish phosphate prodrug of RDV to either bypass the rate-limiting step during translation of the parent nucleoside into its monophosphate, or overcome the low bioavailability due to the inefficient cellular uptake and poor *in vivo* stability (Cho et al., 2012; Elsharif and Dakhil, 2017; Siegel et al., 2017). *In vivo*, two enzymatic activation steps remove the masks to release the nucleoside monophosphate (RDV-MP), in which the ubiquitous esterases (cathepsin A/carboxylesterase1) and phosphoramidases (HINT1-3) are involved (Williamson et al., 2020; Yan and Muller, 2020). Meanwhile, some RDV is hydrolyzed to its parent nucleoside (GS-441524) (Yan and Muller, 2020). Subsequently, both the monophosphorylated RDV and the parent nucleoside are converted to diphosphate and triphosphate by natural kinases. Due to metabolic competition with natural nucleotides such as AMP, ADP, and ATP, RDV inevitably results in perturbation of endogenous RNs, which could restrict the synthesis of viral RNA in turn (Boccardo and Accotto, 1988; Fahima et al., 1993). However, to date, it remains uncertain how RDV exposure affects cellular nucleotides.

Besides alteration of adenine nucleotides, RDV might change the levels of other nucleotides by affecting enzymes in nucleotide synthesis and metabolism. Previous studies have shown that guanine analogues, ribavirin, and 5-ethynyl-1-beta-D-ribofuranosylimidazole-4-carboxamide (EICAR), depleted the GTP pool through inhibition of inosinate dehydrogenase (Stridh, 1983; Balzarini et al., 1993). Similarly, RDV as an adenine analog was hypothesized to inhibit S-adenosylhomocysteine (SAH) hydrolase and adenylate kinase, consequently interfering with the biosynthesis of adenine derivatives (Bistulfi et al., 2009; De Clercq, 2019). Moreover, Kim et al. have reported previously that SARS coronavirus may require more ATP to promote stable helicase translocation necessary for delicate RNA replication (Jang et al., 2020). Endogenous RNs and dRNs pools also affect the response of RDV against viral infection because the disturbance of adenine derivatives will affect the function of RdRps (Vander Heiden and DeBerardinis, 2017). Furthermore, unbalanced changes in dRNs caused by RDV could induce potential side effects because of failure to maintain the dNTPs level causing genetic abnormalities or cell death (Mathews, 2014). This has already been proven that adaptive metabolic reprogramming of RNs and dRNs pools could promote chemotherapy at the early stage of treatment (Brown et al., 2017). Thus elucidation of the disturbances of RDV treatment on RNs and dRNs pool sizes will not only permit us to understand the exact mechanism of action of RDV, but also enhance the antivirus activity based on the targeted-regulation of RNs and dRNs.

So far, there has been no report on the effects of RDV on RNs and dRNs pool sizes due mainly to the difficulty of quantifying these pool sizes, particularly for the monophosphate and diphosphate nucleotides. Recently, we described a simpler, selective and highly sensitive HPLC-MS/MS method for quantification of RNs and dRNs pools in cells after trimethylsilyl diazomethane (TMSD) derivatization (Li et al., 2019). In the present study, the effects of RDV incubation over different timeperiods on RNs and dRNs pool sizes of human bronchial airway epithelial cells (BEAS-2B cells) were investigated using our well-established HPLC-MS/MS methodology. Furthermore, the influence of RDV on cell cycle, RNA and DNA synthesis and protein expression were studied. The results obtained from this study should facilitate understanding the action mechanisms of RDV and assessment of its efficacy and toxicity for developing individualized therapy.

## Materials and Methods

### Reagents and Chemicals

3-(4,5-dimethylthiazol-2-yl)-2,5-diphenyltetrazolium bromide (MTT), dimethyl sulfoxide (DMSO), paraformaldehyde, propidium iodide (PI), and 0.05% RNase A were provided by Sigma-Aldrich Inc. (St. Louis, MO, United States). RDV was purchased from Manhey Chemical Limited (Hong Kong, China). For our experiments, the stock solution of RDV was prepared in DMSO, stored at −20°C, and serially diluted in Dulbecco’s Modified Eagle’s Medium (DMEM) when needed. The final DMSO concentration did not exceed 0.1% throughout this study. 5-ethynyl uridine (EU) and 5-ethynyl-2′-deoxyuridine (EdU) were supplied by Tokyo Chemical Industry Co., Ltd. (Shanghai, China). 4′, 6-Diamidino-2-phenylindole (DAPI) and Alexa Fluor™ 594 were purchased from Invitrogen Co. (Carlsbad, CA, United States). Glycine, Tris, CuSO_4_, ascorbic acid, EDTA, Triton™ X-100, and TWEEN^®^20 were also obtained from Sigma-Aldrich Inc. RIPA buffer (Cell Signaling Technologies Inc. Beverly, MA, United States), Bradford reagent (Bio-Rad Laboratory, Hercules, CA, United States), nitrocellulose membrane (Merck Millipore, United States), and the enhanced chemiluminescence reagents (Invitrogen, Paisley, Scotland, United Kingdom) were also used in this study. For cell culture, DMEM, fetal bovine serum (FBS), penicillin-streptomycin solution, phosphate buffer saline (PBS), and 0.25% trypsin-EDTA solution were obtained from GIBCO (Grand Island, NY, United States).

The stable isotope labeled adenosine-^13^C_10_,^15^N_5_-triphosphate (ATP-^13^C_10_,^15^N_5_) and adenosine-^13^C_10_,^15^N_5_-monophosphate (AMP-^13^C_10_,^15^N_5_), other nucleotide standards and ammonium acetate (NH_4_OAc) were purchased from Sigma-Aldrich Inc (St. Louis, MO, United States). TMSD and tetrafluoroboric acid (HBF_4_) were obtained from Alfa Aesar Co. (Ward Hill, MA, United States). The methanol (LC-MS grade) and acetonitrile used for the HPLC-MS/MS analysis were bought from Anaqua Chemical Supply (Houston, TX, United States). Formic acid was bought from Fisher Scientific Co. (Fair Lawn, NJ, United States) and diethyl ether was obtained from Tedia Co. (Fairfield, OH, United States), while acetic acid (AcOH) and 30% ammonium hydroxide aqueous solution (NH_4_OH) were purchased from J. T. Baker Chemical Co. (Phillipsburg, NJ, United States). The solid phase extraction (SPE) cartridges (WAX, 3 cm^3^; 30 mg, 60 μm) was bought from Waters Co. (Milford, MA, United States), and the chromatographic column Sepax GP-C_18_ (2.1 × 150 mm, 1.8 μm) from Sepax Technologies (Newark, DE, United States) was also used. Ultrapure water was obtained on the basis of a Milli-Q Gradient water system (Millipore, Bedford, MA, United States).

### Cell Culture and Colorimetric MTT Assay

Normal human bronchial epithelial cells BEAS-2B were purchased from the ATCC (Manassas, VA, United States). They were cultured in DMEM supplemented with 10% FBS, 100 U/mL penicillin-streptomycin in a humidified incubator at 37°C, and 5% CO_2_. Cell viability was determined by a modified colorimetric MTT assay (van Meerloo et al., 2011). Briefly, BEAS-2B cells in the exponential phase were seeded in a 96-well plate for 24 h at 37°C, then treated with RDV at different concentrations (0–100 μM) for 24, 48, and 72 h, respectively. After the appropriate incubation time, 10 μL MTT solution (5 mg/ml) was added for another 4 h incubation and 100 μL DMSO was dispensed to dissolve formazan crystals before spectrophotometric measurement at 570 nm using a microplate ultraviolet-visible spectrophotometer. Cell viability was calculated as follows: cell viability (%) = (absorbance of the test group/absorbance of the control group) × 100. The IC_50_ value was taken as the concentration that caused 50% inhibition of cell viability and was calculated using GraphPad Prism (Graph-Pad Software, Inc., La Jolla, CA, United States).

### EU and EdU Detection Using Click Chemistry

In order to label and visualize specifically newly synthesized DNA and RNA, the click chemistry method was used. The experiments were conducted based on previous publications with a slight modification (Jao and Salic, 2008; Akbalik et al., 2017). In short, BEAS-2B cells were grown in 6-well plates at 2.0 × 10^5^ cells/well for 24 h and then incubated for 14 h with 1.0 mM EU or 10 μM EdU in the presence or absence of 10 μM RDV. After the labeling, cells were washed with PBS and fixed with 4% paraformaldehyde for 30 min. The fixed cells were neutralized with 2 mg/ml glycine, rinsed with PBS, and stained for 30 min at room temperature with a click reaction buffer including 100 mM Tris, 1 mM CuSO4, 10 μM Alexa594-azide, and 100 mM ascorbic acid. After staining, cells were washed several times by using PBS with 0.5 mM EDTA, 1% TWEEN^®^20, and 0.1% Triton™ X-100, and then stained with 0.5 μg/ml DAPI for 30 min. Finally, the cells were imaged by IncuCyte ZOOM Live-Cell Analysis Platform.

### Cell Cycle Analysis

BEAS-2B cells were seeded in 6-well plates at 2.0 × 10^5^ cells/well, cultured for 24 h, and then treated with/without RDV for 12, 24, and 48 h, respectively. Cells were then harvested, resuspended in ice-cold PBS, and fixed with 70% ethanol at −20°C overnight. The fixed cells were washed again using ice-cold PBS and incubated with 500 μL PI containing 0.05% RNase A for 30 min at room temperature in the dark. Finally, a cell cycle distribution profile was accessed by flow cytometry after the staining treatment. The percentages of cells in G0/G1, S, and G2/M phases were analyzed using ModFit LT software (Verity Sofware House, Topsham, ME, United States).

### Western Blot Assay

BEAS-2B cells were treated with RDV at the indicated concentration for 12, 24, and 48 h, respectively. Then the cells were washed with cold PBS twice and lyzed with RIPA buffer on ice. The lysates were centrifuged at 12,000 g for 30 min at 4°C in order to acquire the protein samples. The concentration of cellular total protein was measured by using the Bradford reagent at 595 nm according to the manufacturer’s instructions. 30 μg protein samples were loaded on 10% SDS-PAGE gel and transferred onto nitrocellulose membranes. The membranes were blocked with 5% skim milk for 1.5 h, followed by the incubation of primary antibodies diluted in Tris-buffered saline with a Tween^®^ 20 (TBST) buffer (1:1,000 for β-tubulin, ribonucleotide reductase subunit M1 (RRM1), ribonucleotide reductase subunit M2 (RRM2), and p53-controlled ribonucleotide reductase ((p53R2), Cell Signaling Technologies, Danvers, MA, United States) overnight at 4°C. After that, the membranes were washed with TBST and incubated with secondary horseradish peroxidase-conjugated antirabbit IgG antibody (Cell Signaling Technologies, Inc. Danvers, MA, United States) for 1 h at room temperature. The immunoreactive protein bands were finally detected with an Amersham Imager 600 Western blotting system. Densitometry analysis of protein band was performed by Quantity One software (Version 4.6.2, Bio-Rad, United States).

### Sample Preparation and HPLC-MS/MS Analysis

BEAS-2B cells were plated in 10 cm Petri dishes and cultured with medium for 24 h before treatment with RDV. The seeded cell number for 12, 24, and 48 h RDV treatments were 2.5 × 10^6^, 2.0 × 10^6^ , and 1.5 × 10^6^ cells/dish, respectively. After that, the cells were resuspended with ice-cold PBS. The number of cells was counted before centrifugation at 1,200 rpm for 5 min, and the cell pellet was washed with 1.0 ml ice-cold PBS again and centrifuged at 1,200 rpm for 5 min. Subsequently, cell pellets were treated with 150 μL 80% methanol containing 4 µM AMP-^13^C_10_,^15^N_5_ and 2 µM ATP-^13^C_10_,^15^N_5_ as an internal standards (IS). The following sample preparation and the determination of endogenous RNs and dRNs were performed based on the method previously described (Li et al., 2019). The concentrations of cellular nucleotides were finally calculated according to dividing the absolute amount of each RN and dRN in each sample by the corresponding cell number.

### Statistics Analysis

Data analyses were performed using GraphPad Prism software and values were expressed as mean ± standard deviation (SD) from three independent replicate experiments. The statistical significance of the comparison between control and treated groups was determined by Kruskal–Wallis tests. Statistical significance is indicated as *p < 0.05 and **p < 0.01.

## Results

### Remdesivir Decreased the Viability of BEAS-2B Cell Line

**FIGURE 1:**
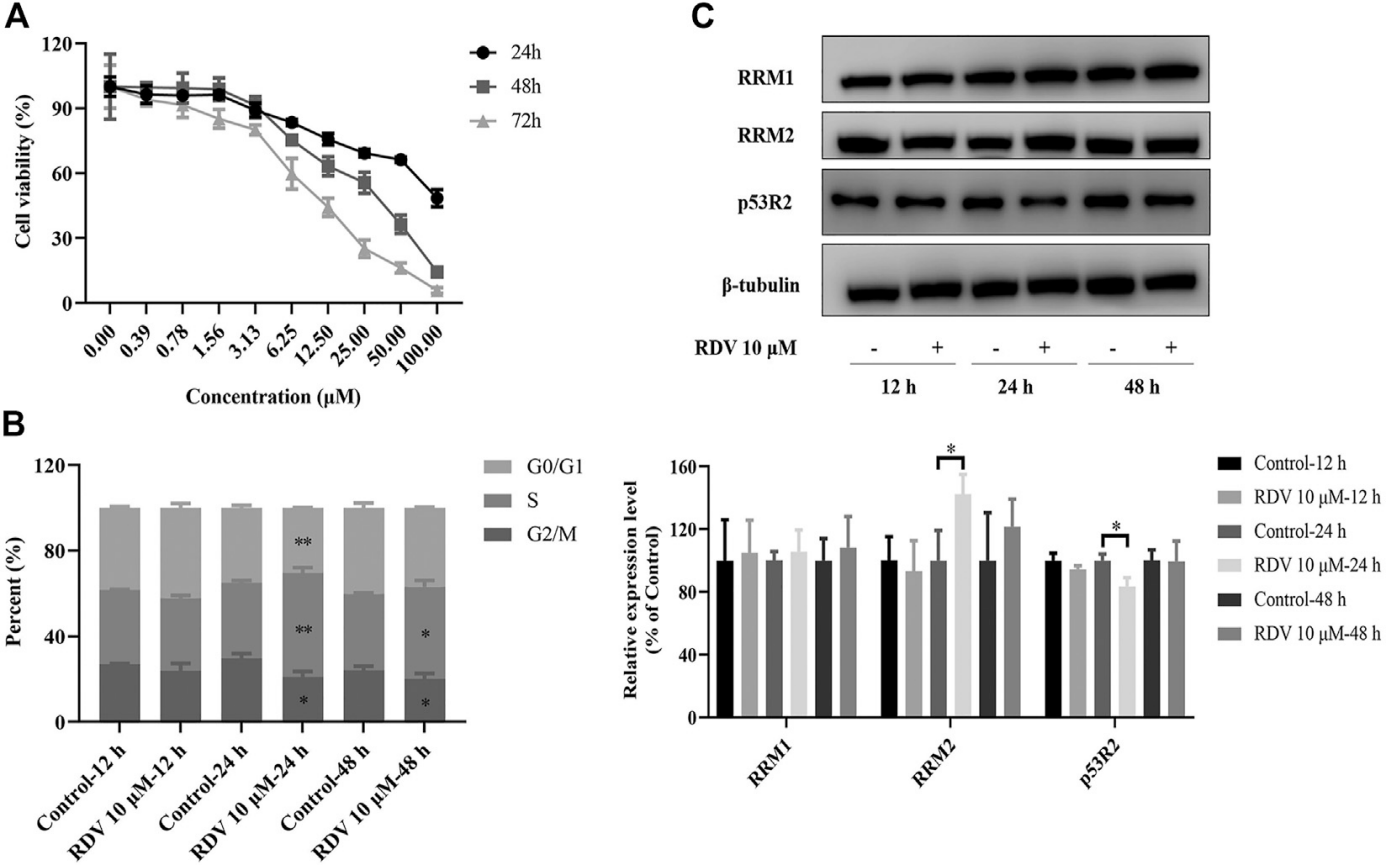
Effects of RDV on **(A)** cell viability **(B)** cell cycle and **(C)** riboreductase expression in BEAS-2B cells treated with 10 μM RDV (RDV, remdesivir; RRM1, ribonucleotide reductase subunit M1; RRM2, ribonucleotide reductase subunit M2; p53R2, p53-controlled ribonucleotide reductase; ∗: *p* < 0.05; ∗∗: *p* < 0.01, compared with control group).

At the beginning of this study, we investigated the cytotoxicity of RDV on BEAS-2B cells using MTT assays. The cells were treated with RDV at various concentrations (0–100 μM) for 24, 48, and 72 h. Figure 1A shows that the cell number gradually decreased as the concentration of RDV increased in all the time points of incubation. The viability of cells presented a dose- and time-dependent reduction. The calculated IC_50_ values in 48 and 72 h were 25.3 ± 2.6 and 9.6 ± 0.7 μM, respectively. 10 μM was chosen for the subsequent experiments.

### Remdesivir Induced S Phase Arrest in BEAS-2B Cells

Based on its significant inhibitory effect on cell viability and proliferation, we investigated the effect of RDV treatment on the distribution of cells in cell cycle at different time points. BEAS-2B cells were treated with or without 10 μM RDV for 12, 24, and 48 h and analyzed by flow cytometry. As shown in Figure 1B, an altered pattern of cell cycle was observed in BEAS-2B cells exposed to 10 μM RDV compared to control. With increase in incubation time, the proportion of cells in S phase significantly increased while the percentage of cells in G2/M phases obviously decreased in comparison to untreated cells. After incubation for 24 h, the percentage of cells in S phase was 35.3 ± 0.75% in control, which gradually increases to 48.69 ± 1.8% in the RDV group (*p* < 0.01). The number of cells in G2 phase decreased from control from 29.67 ± 1.59% to 20.81 ± 1.92% of RDV (*p* < 0.05). Similar results at 48 h were obtained. In summary, RDV could arrest the cells in S phase.

### Remdesivir Inhibited RNA and DNA Synthesis

**FIGURE 2:**
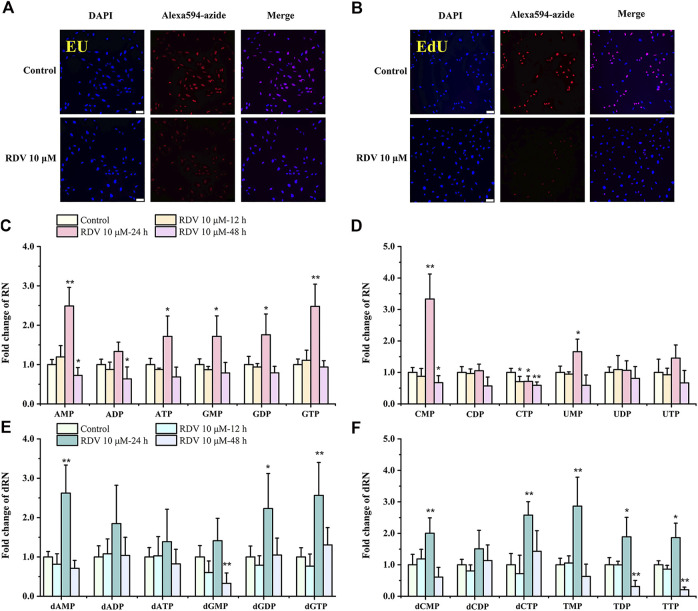
Effects of RDV on RNA and DNA synthesis **(A–B)**, and on nucleotide pools **(C–F)**. Fluorescence microscope images (scale bars = 100 μm) of EU or EdU-mediated click chemistry indicated that RDV treatment for 14 h inhibited the synthesis of **(A)** RNA and **(B)** DNA. Fold changes in nucleotide abundances, as measured by LC/MS-MS, in 10 μM RDV-treated or vehicle-treated BEAS-2B cells for 12, 24 and 48 h **(C–F)** (RDV, remdesivir; EU, 5-ethynyl uridine; EdU, 5-ethynyl-2′-deoxyuridine; RN, ribonucleotides; dRN, deoxyribonucleotides; ∗: *p* < 0.05, ∗∗: *p* < 0.01, compared with control group).

In order to detect the effects of RDV on RNA and DNA synthesis in proliferating cells, we performed EU and EdU staining based on click chemistry. EU and EdU are the structural analogues of uridine and deoxyuridine, respectively. Their triphosphate metabolites compete with UTP and TTP to incorporate into newly synthesized RNA and DNA and subsequently react with azide-modified fluorophores. The fluorescence intensity is proportional to the amount of the incorporated EU and EdU in nascent RNA and DNA. As shown in Figures 2A–B, after incubation with RDV for 14 h, the fluorescence intensity of Alexa594-azide decreased significantly compared to control group, indicating the reduction of RNA and DNA synthesis and the inhibition of proliferation of BEAS-2B cells. Interestingly, not all DAPI stained cells were labeled with EdU. The reason for this phenomenon is that the incorporation of EdU only occurs in S phase during DNA replicating, while DAPI is a nonspecific fluorescent dye with the strong binding ability to the existing or nascent DNA (Qu et al., 2011).

### Perturbation of RNs and dRNs Pool Size by Remdesivir in BEAS-2B Cells

To examine metabolic reprogramming events that influence the cellular response to virus, we used targeted LC/MS-MS via selected reaction monitoring (SRM) to examine changes in the steady-state metabolomic profile of BEAS-2B cells after exposure to 10 μM RDV for 12, 24, and 48 h. The specific nucleotide levels are shown in Supplementary Tables S1, S2. The fold changes of the nucleotides were evaluated by comparison of their concentrations in cells treated with RDV and in the parallel controlled RDV-free cells at the same time points. Significant differences in the metabolite profiles of cells with or without RDV were observed. In general, RDV increased the abundance of the majority of RN and dRN species after 24 h incubation, including a greater than 2-fold increase in AMP, GTP, dAMP, dGDP, dGTP, dCTP, and TMP levels, and then decreased to the normal levels at 48 h (Figures 2C–F). A rational interpretation is that RDV significantly inhibited the synthesis of nascent RNA and DNA, and arrested the cell cycle in S phase, inevitably resulting in the accumulation of (deoxy)nucleoside triphosphates and subsequently the increase of their respective di- and monophosphates (Du et al., 2019). However, it was observed that most of the pyrimidine ribonucleotides remained unchanged or even reduced, among which the significant decrease of CTP was in stark contrast to the 3-fold increase of CMP after incubation for 24 h (Figure 2D).

**FIGURE 3:**
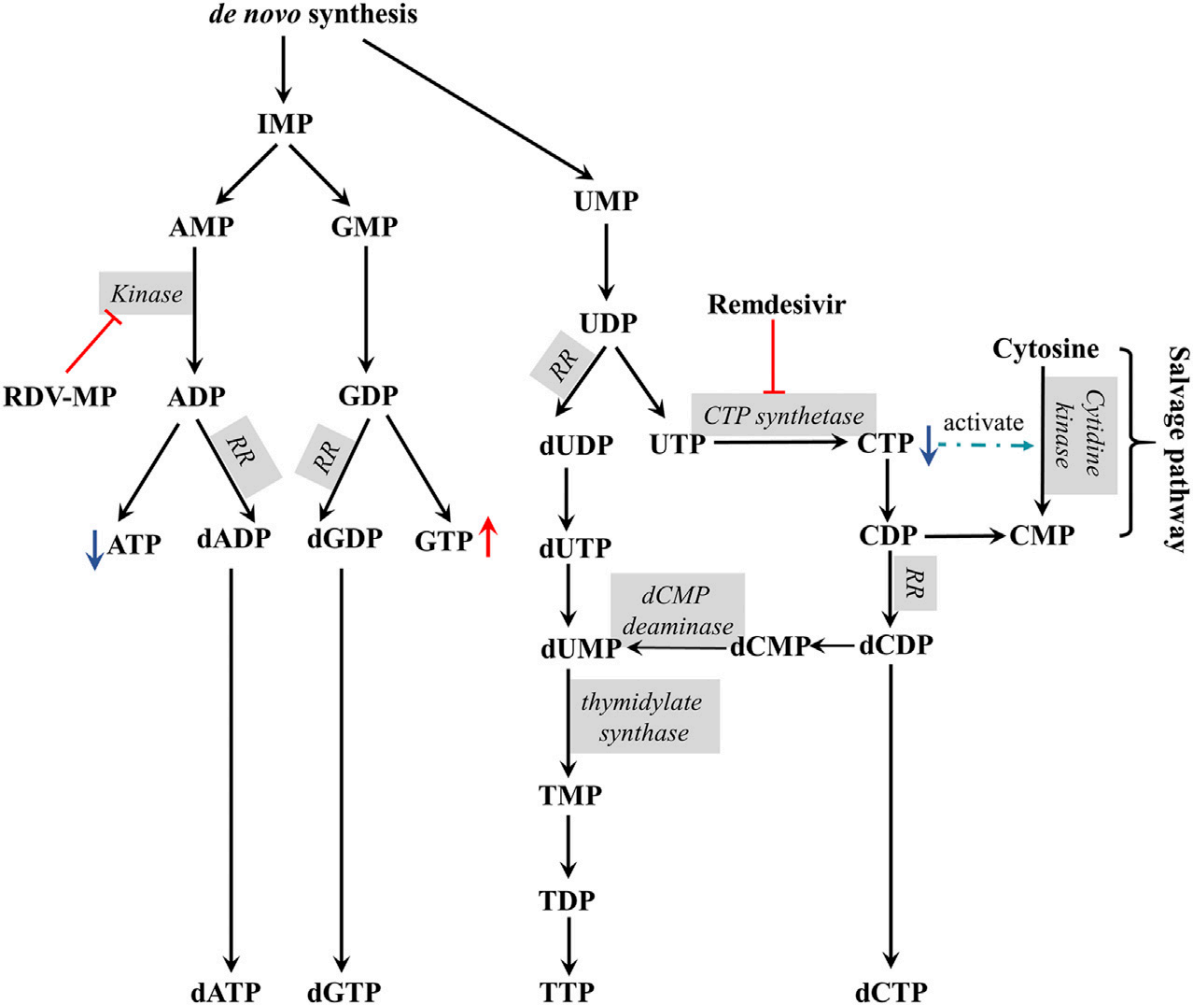
The proposed mechanism of remdesivir induced CTP depletion and the imbalance of other nucleotides in *de novo* and salvage pathways (RR, ribonucleotide reductase).

**FIGURE 4:**
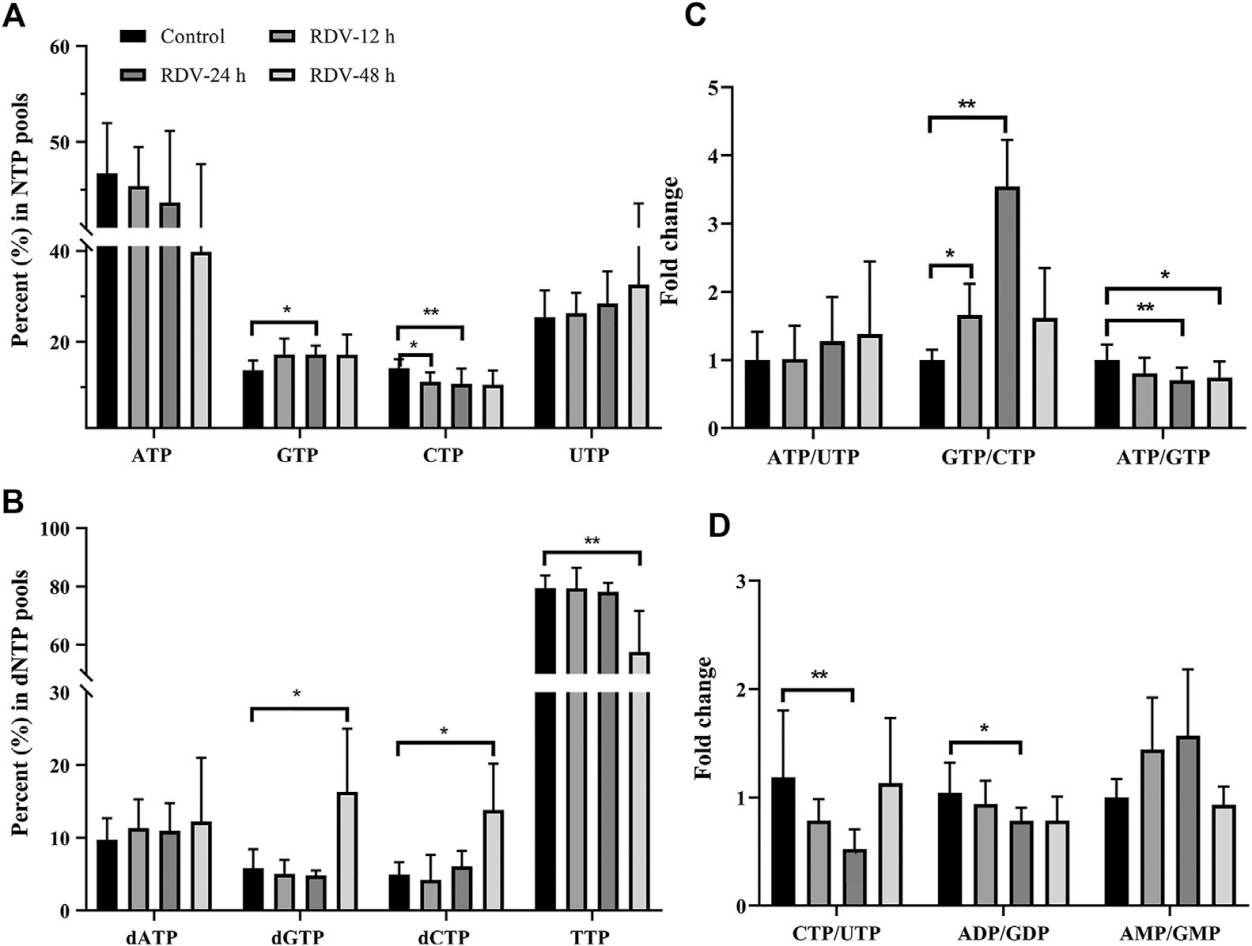
RDV exposure (10 μM) perturbed the balance of **(A)** NTPs and **(B)** dNTPs, and altered the relative ratios of specific nucleotides **(C and D)** (NTP, ribonucleoside triphosphates; dNTP, deoxyribonucleoside triphosphates); ∗: *p* < 0.05; ∗∗: *p* < 0.01, compared with control group.

The possible mechanism of remdesivir-induced CTP depletion and the imbalance of other nucleotides in *de novo* and salvage pathways is shown in Figure 3. CTP is synthesized from UTP by CTP synthase, which is the rate-limiting step of *de novo* CTP biosynthesis and probably a practical target just as in the treatment of leukemia (Verschuur et al., 1998) and parasitic infestations (Hofer et al., 2001; Fijolek et al., 2007; Tamborini et al., 2012). In this study, the ratio of CTP/UTP was calculated showing a significant decrease after 24 h incubation (Figure 4D), implying the inhibition effect of RDV on CTP synthase. Besides the *de novo* pathway, the salvage pathway plays an important role in metabolism of cellular nucleotides too. The relative low level of CTP might allosterically activate the recycle of free bases and nucleosides to promote the production of CMP, resulting in the abnormal elevation of CMP (Figure 3).

The alterations in nucleotide pools were also evaluated by comparing the percent of each NTP in the whole nucleotide pools. It showed that RDV exposure (10 μM) stimulates an increase in GTP and a decrease in CTP (Figure 4A). Consequently, a significant increment of GTP/CTP was observed (Figure 4C), indicating the huge disequilibrium in RN pools. Although there were no statistically significant differences, the ATP level reduced and UTP level increased slightly (Figure 4A), resulting in the elevated ratio of ATP/UTP (Figure 4C). From the aspect of drug disposition, RDV was hydrolyzed to RDV-MP in cell, and furtherly metabolized to RDV-TP. Due to the structural similarity of RDV-MP to AMP, the further phosphorylation of RDV-MP was achieved through the competitive inhibition of adenylate kinase, which inevitably resulted in the accumulation of AMP and the decrease in ADP and ATP. Meanwhile, the accumulation of AMP might inhibit the activity of adenylosuccinate synthase and the whole purine biosynthesis pathway in a negative feedback mode, which would simultaneously decrease the production of GMP, and ultimately GDP and GTP (Nelson et al., 2008). This speculation was proven by the relatively high AMP/GMP ratio and the reduced ATP/GTP and ADP/GDP ratios at 24 and 48 h (Figures 4C–D). The relative percent of dNTPs pools is shown in Figure 4B. The changes in dNTPs percent were contrary to that of NTPs, and there was obvious hysteresis, which was probably because of the allosteric regulation of NTPs to ribonucleotide reductase (RR). In summary, RDV exerted the antiviral activity partly via aggravating the imbalance of nucleotide pools, especially by reducing CTP.

### Remdesivir Upregulated the Riboreductase R2 Expression

The remarkably elevated dNTP pools in cells are probably related to the dNTP synthesis enzymes, especially RR that catalyzes the formation of dRNs from RNs (Mathews, 2006; Liu and Grosshans, 2019). Mammalian RR comprises three subunits including RRM1, RRM2, and p53R2, which are expressed in a cell cycle-dependent manner (Yousefi et al., 2014). In cycling cells, the RRM1 protein is metabolically stable throughout the cell cycle, while the expression and degradation of RRM2 protein limit the S-phase–dependent activity of RR complex, leading to the high cellular dNTPs pools at S phase and low dNTPs pools outside S phase (Engström et al., 1985). In quiescent cells, p53R2 substitutes for protein RRM2 to supply precursor deoxyribonucleotides, which is fundamental to mitochondrial DNA replication and DNA repair. To further investigate whether the growth inhibitory activity of RDV had resulted from the induction of RR, we determined the expression of RRM1, RRM2, and p53R2 by using the western blot assay. Figure 1 C shows that there was no obvious difference of RRM1 level after the BEAS-2B cells incubated with RDV for 12, 24, and 48 h. However, the expression of RRM2 was significantly increased after 24 h exposure to RDV at 10 μM (*p* < 0.05) caused by the S phase arrest. Simultaneously, the p53R2 level presented distinct down-regulated tendency (*p* < 0.05). In addition, there were also no changes in the levels of RRM2 and p53R2 after 48 h incubation with RDV. Taken together, it suggested that RDV inhibited the proliferation of BEAS-2B cells through the impact on RR expression.

## Discussion and Conclusion

Although vaccination is widely considered as the most promising strategy to eliminate COVID-19, virus mutation may be a real threat to the effectiveness of vaccines. SARS-CoV-2 has infected and killed millions of people globally. Before the successful and complete implementation of vaccination for achieving herd immunity, it is urgent to cure infected patients by utilizing the currently available drugs. Among the candidate drugs, RDV was developed as a broad-spectrum antiviral drug, but cannot meet the clinical needs of COVID-19 treatment due to the unsatisfactory therapeutic outcome and high mortality (Beigel et al., 2020). Therefore, it is critical to develop new treatment modalities with high efficacy, among which the combined therapy is a practical strategy.

BEAS-2B was originally established as an immortalized but nontumorigenic epithelial cell line from human bronchial epithelium. The BEAS-2B cell line has been widely used as an *in vitro* cell model in a large variety of studies associated with respiratory diseases including SARS-CoV-2 infection (Wang et al., 2020b). In BEAS-2B, obvious inhibition of biosynthesis of nascent RNA and DNA and arrest of cell cycle in S phase were observed, indicating that remdesivir probably has some negative impact on cell proliferation.

RDV is an adenine nucleotide analog that has been targeted to the process of virus RNA synthesis. In general, nucleotide analogues exert the anticancer or antivirus activity via regulating the activity or expression of the related enzymes in nucleotide synthesis and metabolism pathways to deplete some specific nucleotides and to inhibit the progress of transcription and translation (Nikolaos et al., 2018). Thus, what are the effects of RDV on RNA and DNA synthesis in human cells? Do these effects have any relationship with its efficacy and toxicity? Is it feasible to enhance the antivirus activity of RDV through regulating the related enzymes and metabolites? To our knowledge, no relevant studies have been reported. To answer these questions, the EU staining assay was conducted to evaluate the extent of RDV influence on RNA transcription, and the EdU staining assay to detect the proliferating ability of host cells. As reported, RDV targets the viral RdRps and inhibits RNA chain extension through incorporating the active triphosphate form of RDV into RNA. However, the mechanism of action of RDV on DNA synthesis has not been studied previously.

RR plays a key role in the formation of deoxyribonucleoside diphosphates during DNA synthesis. Experimental results show that RDV inhibited DNA biosynthesis, thus it is rational to investigate the possible effect of RR on DNA synthesis inhibition after RDV treatment. Regulation of RR activity takes place at two levels: through allosteric control of the activity and specificity of RR by nucleoside triphosphate effectors (Nordlund and Reichard, 2006) and by regulation of transcription of the RR genes as a function of the cell cycle (Sun et al., 1992), in response to stresses to the replication machinery, or in response to oxygen content or oxidative stress. Given the structural similarity of RDV diphosphate with the natural ADP, the possible mechanism of RDV affecting RR activity was competitive inhibition by ribonucleoside diphosphate. However, after comparing the ratios of ribonucleoside diphosphate to the corresponding deoxyribonucleoside diphosphate in RDV and control groups (data was not shown), no significant change was observed, indicating that the inhibition of RDV on RR activity was insignificant. Meanwhile, RR expression was investigated. Commonly, RRM1 expression remains relatively constant in actively proliferating cells, while RRM2 expression is controlled by cell cycle. The synthesis of RRM2 starts when DNA replication forks are initiated and goes to a maximum in S phase (Gon and Beckwith, 2006). In this study, the expression of RRM2 increased rather than decreased after 24 h RDV exposure. The probable reason might be the delayed hydrolysis of RRM2 due to DNA replication arrest in S phase. p53R2 gene expression occurs mainly in nonproliferating cells. In postmitotic mammalian cells, protein p53R2 substitutes for protein RRM2, as a subunit of ribonucleotide reductase, and is a prerequisite for mitochondrial DNA replication and DNA repair (Pontarin et al., 2012). Due to the higher proportion of cells arrested in S phase, it is rational for the lower p53R2 level in RDV group at 24 h than in the corresponding control group.

According to the results of the changes of nucleotide, especially the abnormal elevation of CMP and the significant imbalance between CTP with GTP or UTP, we preliminarily speculated that RDV or its metabolites 1) upregulated the biosynthesis of CMP in the salvage pathway, and 2) inhibited the conversion of UTP into CTP by CTP synthase in the *de novo* pathway. In order to clarify these issues, the effect of RDV on purified uridine-cytidine kinase (UCK) and CTP synthase should be further researched. Additionally, as a potential target for RDV treatment, CTP synthase attracted our attention. In the clinical applications of antiviral nucleoside analogues, combination therapy is potent for overcoming drug resistance, such as lamivudine plus adefovir (Yatsuji, et al., 2008; Cai et al., 2016), lamivudine and zidovudine (Mandelbrot et al., 2001), and tenofovir DF plus stavudine (Gallant et al., 2004). Similarly, it was reported that zidovudine could increase the radiosensitizing effects of (E)-2′-Deoxy-(fluoromethylene)cytidine (FMdC) by regulating the alteration of dNTP pools in FMdC treatment. Here, we proposed a combined strategy targeting the metabolic vulnerability sites to enhance RDV’s antiviral efficacy. Cyclopentenyl cytosine, a CTP synthase inhibitor (Kang et al., 1989), has broad-spectrum antiviral activity (De Clercq et al., 1991; Clercq, 1994). Leflunomide, as an inhibitor of *de novo* pyrimidine synthesis (Rückemann et al., 1998), possesses antiviral and immunosuppressive activities (Chong et al., 2006). Based on the action mechanism and the results of nucleotides induced by RDV, a co-administration of RDV with cyclopentenyl cytosine or leflunomide might be a powerful approach and deserves further study.

## Data Availability

The original contributions presented in the study are included in the article/Supplementary Material; further inquiries can be directed to the corresponding authors.

## Abbreviations

RDV, Remdesivir; SARS-CoV-2, Severe acute respiratory syndrome coronavirus 2; COVID-19, Coronavirus disease 2019; RdRps, RNA-dependent RNA polymerases; RR, Ribonucleotide reductase; RRM1, Ribonucleotide reductase subunit M1; RRM2, Ribonucleotide reductase subunit M2; p53R2, p53-controlled ribonucleotide reductase; RDV-MP, Remdesivir nucleoside monophosphate; RNs, Ribonucleotides; dRNs, Deoxyribonucleotides; NTPs, Ribonucleoside triphosphates; dNTPs, Deoxyribonucleoside triphosphates; AMP, Adenosine monophosphate; GMP, Guanosine monophosphate; IMP, Inosine monophosphate; UMP, Uridine monophosphate; CMP, Cytidine monophosphate; ADP, Adenosine diphosphate; GDP, Guanosine diphosphate; UDP, Uridine diphosphate; CDP, Cytidine diphosphate; ATP, Adenosine triphosphate; GTP, Guanosine triphosphate; CTP, Cytidine triphosphate; dAMP, Deoxyadenosine monophosphate; dGMP, Deoxyguanosine monophosphate; dUMP, Deoxyuridine monophosphate; TMP, Thymidine monophosphate; dCMP, Deoxycytidine monophosphate; dADP, Deoxyadenosine diphosphate; dGDP, Deoxyguanosine diphosphate; dUDP, Deoxyuridine diphosphate; TDP, Thymidine diphosphate; dCDP, Deoxycytidine diphosphate; dATP, Deoxyadenosine triphosphate; dGTP, Deoxyguanosine triphosphate; dUTP, Deoxyuridine triphosphate; TTP, Thymidine triphosphate; dCTP, Deoxycytidine triphosphate; HPLC-MS/MS, High-performance liquid chromatography− tandem mass spectrometry.
