# Supplementary material for: Profiling Ribonucleotide and Deoxyribonucleotide Pools Perturbed by Remdesivir in Human Bronchial Epithelial Cells
Source: Front Pharmacol. 2021 May 4;12:647280. doi: 10.3389/fphar.2021.647280 (PMC8120990; doi:10.3389/fphar.2021.647280)
Supplement: Supplementary file 1 [file datasheet1.docx]

Supplementary Table 1. Levels of RNs in Beas-2B cells before and after incubation with remdesivir (mean± standard deviation, pmol/10^6^ cell, n = 5).

|  | Control-12 h | 10 μM-12 h | Control-24 h | 10 μM-24 h | Control-48 h | 10 μM-48 h |
| --- | --- | --- | --- | --- | --- | --- |
| ATP | 3848.2±532.3 | 3367.2±118.9 | 3092.0±341.2 | 5301.6±1437.0* | 4449.9±1102.5 | 3052.5±988.8 |
| ADP | 363.1±77.6 | 318.1±60.8 | 221.7±45.6 | 295.5±46.7 | 281.5±34.6 | 179.3±76.3* |
| AMP | 136.0±30.6 | 162.4±35.0 | 65.0±15.4 | 161.7±27.4** | 162.2±18.9 | 117.7±28.9* |
| GTP | 1159.9±345.0 | 1287.4±265.3 | 834.3±104.8 | 2069.0±421.7** | 1338.8±291.1 | 1254.3±190.9 |
| GDP | 40.7±7.6 | 38.1±3.2 | 42.4±6.5 | 74.6±20.0* | 40.3±5.1 | 31.8±6.1 |
| GMP | 12.4±1.6 | 10.8±0.9 | 8.2±1.9 | 14.0±3.8* | 14.5±3.8 | 11.4±3.5 |
| CTP | 1178.8±238.6 | 836.7±172.4* | 1754.5±200.0 | 1252.7±267.3* | 1300.8±456.7 | 766.2±127.1 |
| CDP | 34.8±5.7 | 33.6±4.6 | 46.6±8.9 | 49.0±8.8 | 57.7±24.1 | 33.0±14.6 |
| CMP | 9.8±3.5 | 8.6±2.2 | 4.7±1.3 | 15.6±3.3** | 20.8±2.9 | 14.0±4.1* |
| UTP | 2124.8±585.9 | 1970.1±392.8 | 2338.8±459.7 | 3401.2±876.3 | 3988.4±1316.1 | 2662.9±1404.9 |
| UDP | 214.6±86.6 | 234.4±85.1 | 206.02±32.4 | 220.3±55.3 | 281.6±105.8 | 227.9±94.6 |
| UMP | 16.2±2.9 | 15.3±1.1 | 16.8±3.5 | 27.8±6.0* | 29.4±9.4 | 17.4±8.6 |
| Energy  Charge | 0.93±0.00 | 0.92±0.01 | 0.95±0.01 | 0.94±0.01 | 0.94±0.01 | 0.94±0.01 |

Note: Each data point is reported as mean ± standard deviation values. (*P < 0.05, **P < 0.01, compared with the control group).

Supplementary Table 2. Levels of dRNs in Beas-2B cells before and after incubation with remdesivir (mean± standard deviation, pmol/10^6^ cell, n = 5).

|  | Control-12 h | 10 μM-12 h | Control-24 h | 10 μM-24 h | Control-48 h | 10 μM-48 h |
| --- | --- | --- | --- | --- | --- | --- |
| dATP | 10.4±3.2 | 10.7±4.6 | 9.9±2.1 | 13.7±7.3 | 7.1±2.2 | 5.9±2.4 |
| dADP | 13.6±3.4 | 14.7±4.6 | 14.0±5.8 | 25.9±12.2 | 12.9±3.1 | 13.4±5.3 |
| dAMP | 11.3±4.5 | 9.2±2.7 | 4.6±0.6 | 12.0±2.9** | 6.5±1.9 | 4.6±1.2 |
| dGTP | 6.1±2.2 | 4.7±1.7 | 2.2±0.7 | 5.7±1.67** | 5.0±1.0 | 6.5±2.2 |
| dGDP | 3.8±0.9 | 3.0±0.8 | 2.3±0.7 | 5.2±1.9* | 3.0±1.3 | 3.1±1.2 |
| dGMP | 2.2±0.7 | 1.3±0.6 | 1.0±0.3 | 1.4±0.5 | 3.1±0.9 | 1.0±0.7** |
| dCTP | 5.4±1.9 | 3.9±2.8 | 2.6±0.8 | 6.7±1.0** | 5.3±2.4 | 7.6±3.1 |
| dCDP | 2.6±0.8 | 2.1±0.4 | 4.6±0.7 | 7.0±2.4 | 4.5±1.9 | 5.1±2.0 |
| dCMP | 2.5±0.9 | 3.0±0.7 | 2.7±0.8 | 5.4±1.2** | 9.2±3.3 | 5.6±2.6 |
| TTP | 84.9±9.9 | 73.1±9.3 | 49.5±6.0 | 92.1±20.4* | 157.1±58.4 | 32.6±11.9** |
| TDP | 46.3±9.5 | 46.3±4.7 | 26.0±4.6 | 49.2±14.4* | 69.1±17.7 | 21.3±12.0** |
| TMP | 18.6±3.5 | 19.6±3.8 | 13.0±2.7 | 37.3±10.7** | 31.4±9.2 | 19.8±10.9 |

Note: Each data point is reported as mean ± standard deviation values. (*P < 0.05, **P < 0.01, compared with the control group).
